# Medical and Philosophical Intelligence

**Published:** 1815-12

**Authors:** 


### MEDICAL AND PHILOSOPHICAL INTELLIGENCE.

*To the Editors of the London Medical and Physical Journal.*

GENTLEMEN,

LEST it should be imagined that I mean to observe a disrespectful silence towards Dr. Kinglake's letter on "topical cold in gouty inflammation," I beg leave to state, through the medium medium of your next Number, that I have no such intention; but that, in consequence of the Journal for this month not arriving until yesterday, and my time being, at present, much occupied by a pressure of professional duties, I fear it will not be in my power to make my reply by the time required for communications intended to appear the succeeding month. I pledge myself, however, to take the earliest opportunity of doing so. I am,

Your very humble Servant,

*Langport; Nov.* 6, 1815. N. W.

---

On the last day of the last month, the Society for the Relief of Widows and Orphans of Medical Men in and about the Metropolis held their anniversary dinner. His R. H. the Duke of Kent was in the chair, and acquitted himself with that ability which might be expected from his ready elocution, and the frequency with which he condescends to preside at similar institutions. When he left the room, Dr. Baillie, who had been appointed to succeed their late worthy president, Mr. Ware, assumed the chair of course. Whether the company were unprepared for the event, which, on that account, was still more grateful to them, or whether the enthusiasm of the moment was greater than the president had prepared himself for, it certainly overcame his feelings in a manner which does infinite honour to his heart. It was some time before the plaudits sufficiently subsided to afford him a hearing; and, when all were mute, the effect was not less remarkable on one who, for more than twenty years, had been a public orator.

The sight of many who had been his scholars, of others his seniors, and some his equals in age, all assembled for so benevolent a purpose, and all, at the same time, directing their eyes towards him, rendered his eloquence that of true nature, and, of course, produced a sympathetic effect on all present.

"Thro' every breast the soft infection ran."

To attempt giving even the substance of what he said, would be alike unfair and impossible. Its influence was such as we have described, and remained, with different degrees, in all present, throughout the whole of the evening.

The meeting was more respectably attended, and more numerous, than on any former occasion; and the enthusiasm in favour of the charity was wrought to such a height, as must produce the strongest and most lasting effects on all present, and in their diligence to enlist others in so laudable a cause.

---

Some of our readers have expressed their wish to see more of continental medicine than our pages have lately contained. We can assure them that no want of diligence on our part has been the cause of this difference. That our neighbours are good experimentalists, and successful in natural history, cannot be questioned. But, respecting the great object of our labours, practical improvements in the healing art, we could almost fancy their progress

 retrograde.

retrograde. Had we room, like some of our brother journalists, for all their little pithy suggestions, or could we impose them on our subscribers, it would save us a world of trouble in rejecting what we submit to read, and in searching for more useful matter. To explain ourselves better, we shall copy the following from a contemporary journal.

"*Royal Institute of France.*—M. Magendie's fine experiments on vomiting will be recollected, and the invitation by the Class to examine the part which the œsophagus may have in this disorderly movement of the stomach. Though these researches have not yet led to a decisive result, they appeared to him sufficiently interesting to be communicated.

"The alternate contractions and relaxations of the œsophagus appear to him to take place only in the lowest third of it, where it is chiefly excited by the nerves of the eighth pair. The contraction increases much, and continues a long time, when the stomach is full. When the œsophagus is cut and detached from the diaphragm, the injection of tartar emetic into the veins does not produce vomiting: its introduction into the stomach becomes necessary.

"M. Delpech, Professor of Surgery at Montpellier, has sent a memoir to the Class on the hospital sore, a kind of gangrene which affects the sores when the wounded patients are too numerous. He has ascertained that this dreadful malady, of which few practitioners have spoken, is produced by a local contagion. It is propagated by the linen, the charpee, and the instruments. Its progress is slower when the patients can be exposed to a current of air. The most minute attention to cleanliness is necessary to prevent it from spreading. But the only true remedy, according to M. Delpech, is the application of the actual cautery to the parts affected with it.

"Some years ago, M. Maunoir, surgeon in Geneva, sent a memoir on the advantages of the method of amputation invented in England, and which consists in cutting the skin lower down than the bone and the muscles, so as to preserve a sufficient quantity to cover the stump, by bringing it immediately in contact.

"M. Roux, surgeon at Paris, has presented a memoir on the same subject, in which he has shewn, from his own experiments, that this method diminishes the sufferings of the patient, that it prevents hæmorrhages and suppuration, that it greatly accelerates the cure of the sore, and that it leaves the stump in a more convenient state, and subject to fewer accidents. He points out the precautions necessary to avoid some inconveniences ascribed to it by those who performed it ill, and particularly to afford the blood and pus, if any be formed, a sufficient passage. M. Percy, our associate, who employed it since his youth, and who, as he informs us himself, has had the melancholy advantage of amputating more limbs than perhaps any surgeon that ever existed, expresses strongly in his report his wish that the memoir of M. Roux may soon render so useful a process general.

 "Two

"Two young surgeons of Paris, MM. Lisfrand and Champenne, have made known their method of amputating the arm at its upper joint, one of the most difficult operations in the surgical art. By making the instrument penetrate under the two eminences of the *omoplate*, called acromion and coracoid process, they reach directly the capsule of the joint, and terminate the operation more quickly than by any of the methods employed before them.

"M. de Saissy, surgeon at Lyons, has cured several deaf people by injections into the cavity of the tympanum, through the tube of Eustachius. He has sent to the Class an account of his method, and the history of the cures which he has performed."

Mr. Magendie's fine experiments we should call unnecessarily cruel. After cutting off the abdominal muscles of some living animals, cutting out the diaphragms of others, and the stomachs of others, he has proved what Mr. Hunter remarked without a single mutilation, and in a much more satisfactory manner, inasmuch as the animals were in their natural condition. (Animal Economy, p. 199, 2d edit.)

We trust the interesting prize essay of M. Brugmans will be a sufficient apology for our omitting any notice of several minor productions concerning hospital gangrene.

M. Maunoir's operation, renewed by M. Roux, we need not say, has been the practice of England for more than thirty years, nor is there one of our lately numerous juvenile correspondents, who has been twelve months in the shop, that can be ignorant of it.

M. Saissy's mode of curing deafness is older than either of the above-mentioned discoveries, and nothing but its uncertainty, and the frequency of its failure, has prevented its general adoption in England. Perhaps we might add the difficulty with which it is attended, and the doubts of some whether it has ever been accomplished in a satisfactory manner.

---

The Hermaphrodite in Paris, to which we alluded in a former Number, does not materially differ, in the organs of generation, from the subject whose case is related by Cheselden. In the other parts of the body, we have accounts to which we are not disposed to give implicit credit. Female mammæ, and hairs on the legs, add so much to the marvellous, that we shall not anticipate the surprise which may be excited when this unfortunate creature appears in London, which is expected in the course of the winter.

---

It is with much pleasure we announce that the plan of Mr. Young, recommended, in our last, by Dr. Denman, continues to be pursued at the Middlesex Hospital, where a case has lately occurred in which it has exceeded the expectations of the most sanguine, and by which it has acquired the good opinion of the candid part of the most sceptical.

We

We have to lament the loss of the venerable Lettsom, after a very short illness, which assumed the appearance of acute rheumatism, erysipelas, and pleurisy. His complaint is believed to have originated in remaining for two hours in a cold room to examine a dead body. Dr. L. was a native of Tortola, where his family possessed a landed estate; as soon as he became in possession of which, he emancipated all the negroes. He married a lady of considerable property: by her jointure and settlements the three remaining children who survive him are well provided for. He came so early into full practice in the city, that he was generally supposed older than his real age, which was only 71. He lived, however, without any apparent alteration in the faculties of his mind, or the strength of his body.

About forty years ago, under his auspices, the Medical Society of London was erected, and endowed with the house at which they assemble, and which contains their library, to which he was a considerable contributor. There were few charitable or scientific institutions in which his name did not appear, or to the success of which he did not contribute. Though not to be considered among the learned, nor the first scientific men of the age, he was a judicious practitioner, diligent, candid, and successful. Like most of the children of the sun, he was *prodigus æris*, which somewhat embarrassed him, but never induced him to act inconsistently with his character for either justice or generosity. His works, though numerous, were few of them well received; but, if they do not elevate his medical character, they certainly would not disgrace any one in much higher estimation.

He was buried at the Friend's Ground, near Bunhill-row, according to the custom of that sect. Of his surviving children, one son is married to the daughter of the present Attorney-general.

---

As we were making up our Number, we learned the much-lamented death of our venerable correspondent Dr. Denman, who, at the age of 83, apparently free from all the infirmities of advanced life, died, in the night, with so little uneasiness, that his dissolution was not discovered till he was expected to arise in the morning. He has left a widow, who must, for the remainder of her life, feel severely the loss of a mate to whom she has been so long united. She is, however, fortunate in three children, worthy of such parents, and most happily settled. Of the ladies, one is married to Dr. Baillie, and the other to Dr. Croft. Both are mothers. Her son is in the church, with every fair prospect, of preferment, and, which is still more desirable, of being useful in that sacred profession.

### METEOROLOGICAL REGISTER.

*From October the 25th, to November the 25th,* 1815.

Kept by C. BLUNT, Philosophical Instrument Maker, No. 38, Tavistock-Stieet, Covent-Garden.

| Moon. | Day. | Wind. | Barometrical Pressure. Max. | Min. | Mean. | Temperature. Max. | Min. | Mean. | |
|---|---|---|---|---|---|---|---|---|---|
| | 26 | N | 29 50 | 29·46 | 29·487 | 56 | 40 | 47·75 | Rain |
| | 27 | N | 29·60 | 29·48 | 29 515 | 55 | 36 | 45·75 | Rain |
| | 28 | NE | 29·96 | 29 74 | 29·825 | 54 | 36 | 45·25 | Rain |
| | 29 | NE | 29 90 | 29·90 | 29·90 | 55 | 35 | 44·5 | Fair |
| | 30 | NE | 29·87 | 29 86 | 29·867 | 56 | 33 | 42 75 | Fair |
| | 31 | E | 29·99 | 29·96 | 29·967 | 55 | 34 | 42·5 | Fair |
| ● | 1 | N | 29 96 | 29·94 | 29·945 | 55 | 35 | 43· | Fair |
| | 2 | N | 30·07 | 29 96 | 30· | 54 | 32 | 41·5 | Fair |
| | 3 | N | 30·28 | 30·24 | 30·257 | 53. | 27 | 39·25 | Fair |
| | 4 | N | 30·29 | 30·28 | 30·287 | 53 | 30 | 38·5 | Fair, Rain during Nig. Fog in Morn. |
| | 5 | W | 30 21 | 30·21 | 30·21 | 55 | 35 | 43·5 | Fair |
| | 6 | SW | 30 17 | 30·13 | 30·145 | 56 | 37 | 46·5 | Rain |
| | 7 | W | 30·10 | 30·05 | 30 07 | 56 | 32 | 43·75 | Rain |
| | 8 | W | 29·99 | 29·94 | 29 912 | 56 | 32 | 43 25 | Rain |
| ◐ | 9 | W | 29 96 | 29·87 | 29·536 | 55 | 38 | 44·75 | Rain |
| | 10 | SW | 30·11 | 30·08 | 30·09 | 53 | 40 | 46 25 | Fair |
| | 11 | SW | 30·09 | 30·04 | 30·06 | 57 | 49 | 46·5 | Rain |
| | 12 | W | 30·08 | 30·08 | 30 08 | 55 | 41 | 46· | Rain |
| | 13 | W | 29·15 | 29· | 29·102 | 54 | 33 | 43·5 | Rain |
| | 14 | NW | 29· | 29·14 | 29·082 | 52 | 31 | 42 25 | Fair |
| | 15 | N | 29·29 | 29·14 | 29 187 | 50 | 29 | 38·75 | Snow d.Nig. |
| ○ | 16 | N | 29·53 | 29·36 | 29·597 | 49 | 25 | 36·75 | Sleet, Fair |
| | 17 | N | 29 80 | 29·56 | 29·675 | 49 | 23 | 36·5 | Sleet, Fair |
| | 18 | N | 30·02 | 29·91 | 29·957 | 46 | 25 | 34· | Fair |
| | 19 | E | 29·85 | 29 85 | 29·85 | 45 | 25 | 33·5 | Fair |
| | 20 | E | 29·72 | 29 64 | 29 66 | 46 | 30 | 36· | Fair |
| | 21 | E | 29·67 | 29·64 | 29·907 | 46 | 30 | 37· | Fair |
| | 22 | E | 29·94 | 29·75 | 29·842 | 45 | 28 | 35·5 | Fair |
| ◑ | 23 | E | 30·18 | 30 08 | 30·12 | 46 | 28 | 35·25 | Fair |
| | 24 | N | 30·37 | 30·26 | 30·307 | 46 | 28 | 34·75 | Fair |
| | 25 | N | 30·43 | 30·43 | 30 43 | 50 | 32 | 38·75 | Fair |

RESULTS.

Mean barometrical pressure of the month 29·889
Maximum 30·43, wind at N
Minimum 29· ——— W

Mean temperature of the month 38·9783 deg.

Maximum 57, wind at SW

Minimum 23, ——— N

*Scale exhibiting the prevailing Winds during the Month.*

| N | NE | E | SE | S | SW | W | NW |
|---|---|---|---|---|---|---|---|
| 12 | 3 | 6 | 0 | 0 | 3 | 6 | 1 |

| | Mean barometrical pressure. | Mean temperature. |
|---|---|---|
| From the last quarter on the 25th Oct. to the new moon on the 1st of Nov. | 29·76 | 44·75 |
| ——— new moon on the 1st, to the first quarter on the 9th | 30·033 | 42·406 |
| ——— first quarter on the 9th, to the full moon on the 16th | 29·591 | 44· |
| ——— full moon on the 16th, to the last quarter on the 23rd | 29·784 | 35·607 |

MONTHLY

### REPORT OF DISEASES.

During the early part of the month, the mildness of the season produced a corresponding salubrity in the town, and the autumnal complaints having ceased, made way for the exanthemata, which usually prevail in proportion as the atmosphere is free from other epidemics. Small-pox is very frequent and very fatal. How can we account for this inconsiderateness in parents, and in others old enough to know their danger? The hooping-cough has been greatly exasperated by the increased cold, and some phthisical subjects, who seemed to remain stationary for a time, are making rapid advances towards dissolution. Cutaneous diseases, in exposed parts, are re-appearing; and rheumatism, with all its terrors. All the complaints, however, excepting the advanced stages of phthisis, seem mitigated, if not cured, by evacuations. Tight rollers are found very serviceable in rheumatisms of almost every description.

A violent head-ach of long standing, and which yielded to no plan of treatment, not excepting mercury, has been alleviated by nauseating doses of antimony, and frequent cupping on those parts of the scalp where the patient complained most; but the disease still exists, and requires constant and frequent attention. A strongly-marked case of hectica senilis has occurred at a more early period of life than usual. Nothing has succeeded but a more generous mode of living.

